# Fibroblast growth factor receptor 4 (FGFR4) and fibroblast growth factor 19 (FGF19) autocrine enhance breast cancer cells survival

**DOI:** 10.18632/oncotarget.9328

**Published:** 2016-05-12

**Authors:** Kai Hung Tiong, Boon Shing Tan, Heng Lungh Choo, Felicia Fei-Lei Chung, Ling-Wei Hii, Si Hoey Tan, Nelson Tze Woei Khor, Shew Fung Wong, Sze-Jia See, Yuen-Fen Tan, Rozita Rosli, Soon-Keng Cheong, Chee-Onn Leong

**Affiliations:** ^1^ School of Postgraduate Studies, International Medical University, Bukit Jalil, Kuala Lumpur, Malaysia; ^2^ Oral Cancer Research and Co-ordinating Center (OCRCC), Faculty of Dentistry, University of Malaya, Kuala Lumpur, Malaysia; ^3^ Cancer Research Initiatives Foundation, Sime Darby Medical Centre, Subang Jaya, Malaysia; ^4^ Institute of Biological Chemistry, Academia Sinica, Taipei, Taiwan; ^5^ Center for Cancer and Stem Cell Research, International Medical University, Bukit Jalil, Kuala Lumpur, Malaysia; ^6^ School of Medicine, Faculty of Medical and Health Sciences, The University of Auckland, New Zealand; ^7^ School of Medicine, International Medical University, Bukit Jalil, Kuala Lumpur, Malaysia; ^8^ UPM-MAKNA Cancer Research Laboratory, Institute of Bioscience, Universiti Putra Malaysia, UPM Serdang, Selangor, Malaysia; ^9^ Faculty of Medicine and Health Sciences, University Tunku Abdul Rahman, Bandar Sungai Long, Selangor, Malaysia; ^10^ School of Pharmacy, International Medical University, Bukit Jalil, Kuala Lumpur, Malaysia

**Keywords:** fibroblast growth factor, breast cancer, RNAi screen, FGFR4, FGF19

## Abstract

Basal-like breast cancer is an aggressive tumor subtype with poor prognosis. The discovery of underlying mechanisms mediating tumor cell survival, and the development of novel agents to target these pathways, is a priority for patients with basal-like breast cancer. From a functional screen to identify key drivers of basal-like breast cancer cell growth, we identified fibroblast growth factor receptor 4 (FGFR4) as a potential mediator of cell survival. We found that FGFR4 mediates cancer cell survival predominantly via activation of PI3K/AKT. Importantly, a subset of basal-like breast cancer cells also secrete fibroblast growth factor 19 (FGF19), a canonical ligand specific for FGFR4. siRNA-mediated silencing of FGF19 or neutralization of extracellular FGF19 by anti-FGF19 antibody (1A6) decreases AKT phosphorylation, suppresses cancer cell growth and enhances doxorubicin sensitivity only in the FGFR4^+^/FGF19^+^ breast cancer cells. Consistently, FGFR4/FGF19 co-expression was also observed in 82 out of 287 (28.6%) primary breast tumors, and their expression is strongly associated with AKT phosphorylation, Ki-67 staining, higher tumor stage and basal-like phenotype. In summary, our results demonstrated the presence of an FGFR4/FGF19 autocrine signaling that mediates the survival of a subset of basal-like breast cancer cells and suggest that inactivation of this autocrine loop may potentially serve as a novel therapeutic intervention for future treatment of breast cancers.

## INTRODUCTION

Global gene profiling has uncovered previously unrecognized subtype of human breast cancer, including the so-called “triple-negative” or “basal-like” tumors characterized by estrogen/progesterone receptor negativity, lack of HER2 amplification and high frequency of p53 mutation [1–6]. These refractory tumors are therefore insensitive to effective hormonal therapy or herceptin-based therapy and have a poor prognosis compared to other subtypes [1, 7–9].

Although large-scale sequencing of breast cancer genomes has uncovered critical mutations in kinase signaling implicated in basal-like breast cancers for drug development efforts, the functional role of many of these genetic abnormalities remains unclear [10]. It is also plausible that other key modulators of the malignant phenotype might not show DNA sequence alterations. To better understand the role of aberrant kinase signaling and identify bona fide molecular targets for drug development in basal-like breast cancers, we undertook a large-scale loss-of-function shRNA screen of the kinome, leading to the identification of fibroblast growth factor receptor 4 (FGFR4) as an essential kinase critical for the proliferation and survival of basal-like breast cancer cells.

FGFR4 belongs to the FGFR protein family comprising of four highly conserved receptor tyrosine kinase members (FGFR1, 2, 3 and 4) and a kinase domain-deficient member (FGFR5 or FGFRL1) [11–14]. In the presence of cofactors such as heparan sulfate proteoglycans (HSPG), FGFRs interact with a wide range of fibroblast growth factors (FGFs) to promote receptor dimerization, autophosphorylation and activation of signaling pathways governing various biological responses involving cell differentiation, proliferation, metastasis, angiogenesis and apoptosis [11–13, 15, 16]. The importance of FGF/FGFR signaling in tumor pathogenesis has been highlighted in large-scale analyses of human cancer genomes, in which components of the FGF/FGFR signaling pathways were the most commonly amplified or mutated in human cancers [12, 17–20].

To date, most of the understanding on the functional role of FGFRs and their signaling pathways has been derived mainly from the study of FGFR1-3. Only limited studies on FGFR4 have been reported so far. Although all family members share significant homology, there are major differences in terms of FGF binding specificity, activation of downstream signaling pathways and tumor-specific genetic alterations [12, 21]. Unlike FGFR1-3, whose activating mutation plays a central role in tumorigenesis, FGFR4 is rarely mutated in cancer or in other diseases [12, 22]. Although altered expression has been documented in breast, lung, pancreatic and prostate cancers, the specific role for FGFR4 in these cancers is not well established [13].

Here, we demonstrated that FGFR4 is highly expressed in a subset of breast cancer cells and primary tumors. Depletion of endogenous FGFR4 induces tumor-specific lethality in MDA-MB-468 and HCC1937 basal-like breast cancer cells but not in the MCF-7 luminal-like breast cancer cells nor in SKBR3 HER2-positive cells. We showed that survival of MDA-MB-468 and HCC1937 is regulated by a constitutively active FGFR4/FGF19 autocrine signaling, which activates downstream PI3K/AKT signaling. Inhibition of FGF19 by siRNA or a neutralizing antibody induced significant apoptotic cell death in an AKT-dependent manner. Importantly, FGFR4, FGF19 and phospho-AKT were found to be co-overexpressed in a subset of basal-like breast tumors, suggesting that the FGFR4/FGF19 autocrine loop might be of clinical importance. Together, our results implicate that FGFR4 and FGF19 autocrine signaling may serve as a potential therapeutic target for the treatment of refractory basal-like breast cancers.

## RESULTS

### Kinome-wide shRNA library screen identifies 15 candidate kinases important in regulating basal-like breast cancer cells survival

**Figure 1:**
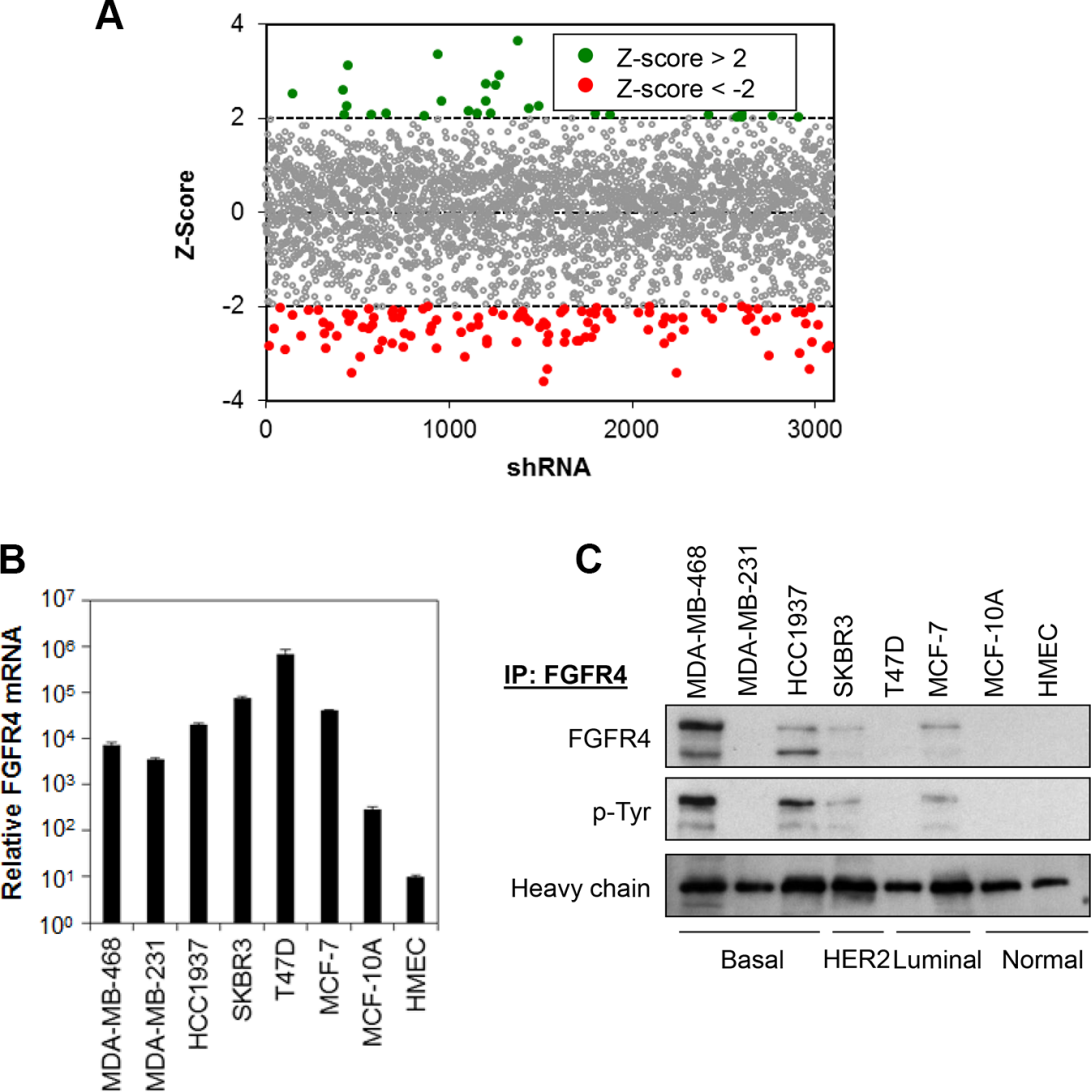
Kinome shRNA library screen identifies FGFR4 as putative target mediating breast cancer cell survival (**A**) Kinase shRNA screen scatter plot. Z-scores are plotted on the y-axis against 3109 corresponding shRNAs on the x-axis. Red circled dots represent shRNA that reduces cell proliferation and green circled dots represent shRNA that induces cell proliferation. (**B** and **C**) FGFR4 is overexpressed in a subset of breast cancer cells. FGFR4 mRNA and protein expression was determined by qPCR and IP-Western blotting, respectively. Heavy chain serves as loading control.

The RNAi Consortium (TRC) kinome shRNA library, consisting of 3109 lentiviruses carrying shRNA sequences targeting 673 human kinase genes, was used to screen for the roles of these kinases in mediating the survival of MDA-MB-468 breast cancer cells. Each gene is represented by at least 3–5 individual constructs, targeting different regions of the gene sequence. As shown in Figure 1A, a total of 116 (3.7%) shRNA constructs targeting 89 kinases in the TRC kinome library were identified to induce significant growth inhibition (Z-score < −2) in MDA-MB-468 cells. Out of the 89 candidate kinases, 15 were further identified as hits, based on the criteria that 1) at least two independent shRNAs targeting a specific gene exhibit a *Z*-score of less than −2, and 2) the *P*-value of Redundant siRNA Activity (RSA) Analysis of each kinase is less than 0.05 (Table 1). Indeed, independent depletion of the endogenous FGFR4, RIPK1, SPHK1, AURKB and PIK3CD significantly reduced MDA-MB-468 cell survival, consistent with the results obtained in the primary screen (Supplementary Figure S1). In addition, several hits, including PLK1 [23, 24], PRKAA1 (or AMPK) [25–27], PIK3CG [28–30] and ERBB3 [31–36] have also been previously shown to mediate cancer cell survival and growth, independently validating the results of our screen.

**Table 1:** Hits identified from the lentiviral shRNA primary screen

Kinases	Description	Hits^1^	Log *P*-value^2^
PLK1	polo-like kinase 1	4/4	−11.40
AURKB	aurora kinase B	4/4	−6.83
PIK3CD	phosphatidylinositol-4,5-bisphosphate 3-kinase, catalytic subunit delta	4/5	−6.20
FGFR4	fibroblast growth factor receptor 4	3/3	−5.37
RIPK1	receptor (TNFRSF)-interacting serine-threonine kinase 1	4/5	−4.69
STK33	serine/threonine kinase 3	2/4	−4.07
SPHK1	sphingosine kinase 1	3/5	−3.83
PLK3	polo-like kinase 3	3/5	−3.83
ERBB3	erb-b2 receptor tyrosine kinase 3	2/9	−3.08
PIK3CG	phosphatidylinositol-4,5-bisphosphate 3-kinase, catalytic subunit gamma	2/5	−3.00
MAP4K3	mitogen-activated protein kinase kinase kinase kinase 3	2/4	−2.33
EPHA6	EPH receptor A6	2/5	−1.85
CDKL2	cyclin dependent kinase like 2	2/5	−1.77
SRPK2	SRSF protein kinase 2	2/4	−1.62
STK3	serine/threonine kinase 3	2/5	−1.59

1 Hits indicates the number of shRNA that exhibits Z-score < −2 over the total number of shRNA targeting the same gene.

2 Log *P*-value as determined by RSA.

### FGFR4 is overexpressed in a subset of human breast cancer cell lines

Since FGFR family proteins have been reported to play a functional role in various cancer types [11, 12], and the functional role of FGFR4 in basal-like breast cancers remains elusive [37], we decided to focus on understanding the mechanism underlying FGFR4-mediated cell survival in breast cancer cells.

We first evaluated whether FGFR4 is expressed in a panel of breast cancer cell lines consisting of basal-like breast cancer cells (MDA-MB-468, MDA-MB-231 and HCC1937), luminal-like breast cancer cells (T47D and MCF7), HER2 amplified breast cancer cells (SKBR3) and non-transformed breast myoepithelial cells (MCF10A and HMEC). Real-time qPCR shows that FGFR4 mRNA is highly expressed, approximately 10- to 2000-fold, in all the breast cancer cells tested as compared to MCF10A and HMEC (Figure 1B). The level of gene expression, however, does not correlate with the protein expression as FGFR4 proteins were only detected in MDA-MB-468, HCC1937, SKBR3 and MCF7 cells, while no FGFR4 protein expression was detected in the MDA-MB-231, T47D, MCF-10A and HMEC cells (Figure 1C). Interestingly, FGFR4 proteins were found to be tyrosine-phosphorylated in cells that express them, suggesting that FGFR4 proteins are constitutively active in these cancer cells.

### Knockdown of FGFR4 induces tumor-specific cell death in MDA-MB-468 and HCC1937 cells

**Figure 2:**
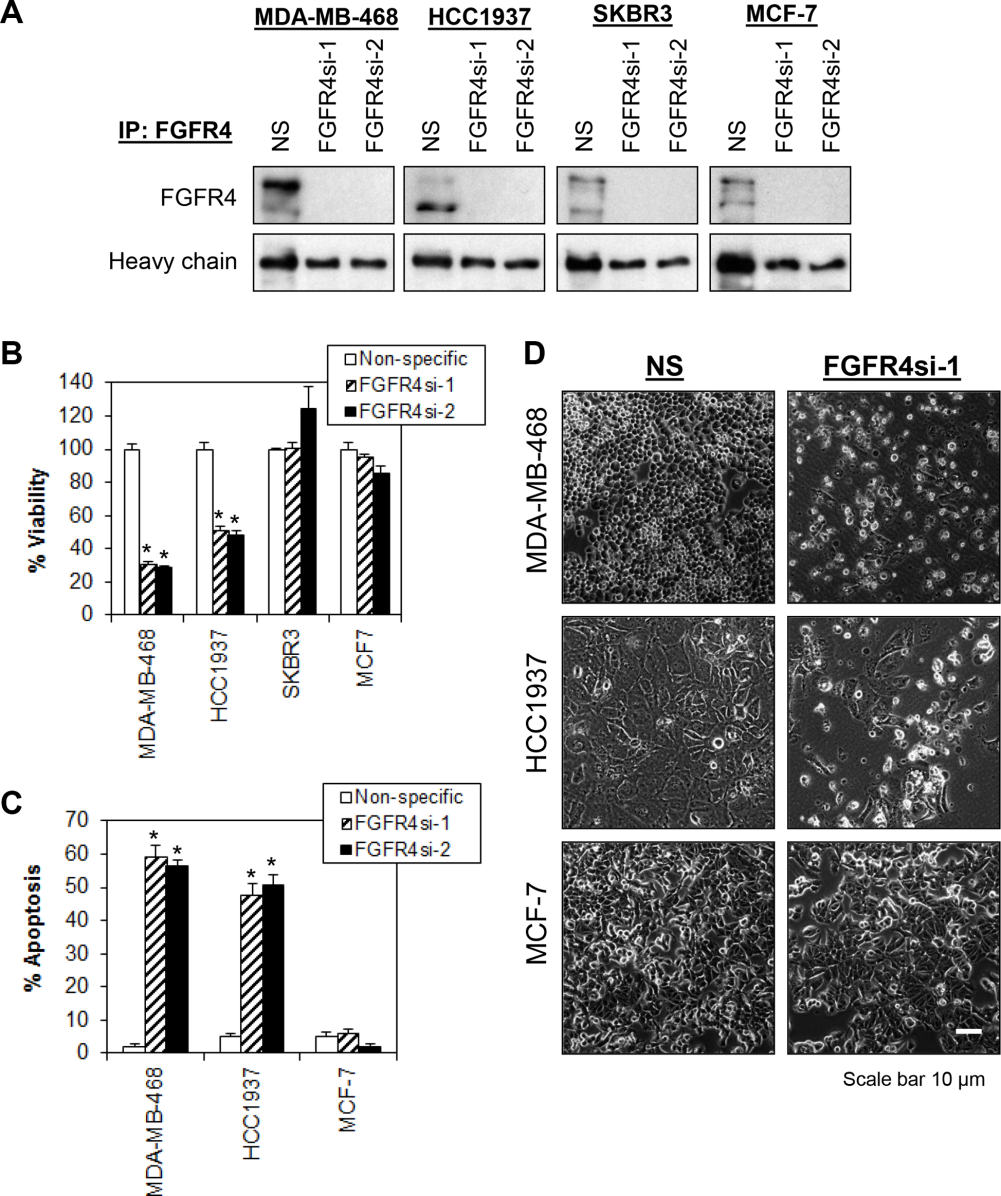
Depletion of endogenous FGFR4 induces tumor-specific cell death in breast cancer cells (**A**) Cells were transduced overnight with a non-targeting lentiviral shRNA (NS) and two independent shRNA targeting FGFR4 (FGFR4si-1 and FGFR4si-2). Lysates were collected at 96 h post-transduction and analyzed by FGFR4 IP-Western blotting. Heavy chain serves as loading control. Note that more than 90% of endogenous FGFR4 were knocked down by independent shRNA as compared to the nonspecific shRNA control. (**B**) FGFR4 depletion selectively inhibits the proliferation of MDA-MB-468 and HCC1937. Cell viability was measured using CellTiter-Glo assay 5 days post-transduction. (**C**) FGFR4 knockdown-induced apoptosis in MDA-MB-468 and HCC1937. Cells were collected 72 h post-transduction and analyzed by annexin V/7-AAD staining. (**D**) Morphological changes following knockdown of FGFR4 in MDA-MB-468, HCC1937 and MCF-7 cells 5 days post-transduction. Original magnification, ×100. Bars represent means ± s.d. of three independent experiments. (*) indicates statistical significance compared with control cells transduced with a non-targeting shRNA (NS) (*P* < 0.01, Student's *t*-test).

To determine whether depletion of endogenous FGFR4 has any effect on the proliferation and survival of breast cancer cells that exhibit constitutively active FGFR4, we performed lentiviral shRNAs-mediated knockdown of FGFR4 in a panel of breast cell lines. Efficient knockdown of FGFR4 in all breast cell lines by two independent shRNA constructs was demonstrated in IP-Western (Figure 2A). Interestingly, only the basal-like MDA-MB-468 and HCC1937 cells exhibit significant reduction in cell proliferation and induction of apoptosis (*P* < 0.01, Student's *t*-test) while no such effect was observed in MCF-7 and SKBR3 that expressed FGFR4, or in cells that have no FGFR4 expression (MDA-MB-231, T47D, MCF10A and HMEC) (Figure 2B–2D). This result suggests that FGFR4 is critical for the survival of basal-like MDA-MB-468 and HCC1937 cells but not for the luminal-like MCF-7 cells or the HER2 amplified SKBR3 cells, which also expressed FGFR4. Hence the regulation of tumor-specific cell survival in different subtypes of breast cancer might be context-dependent.

### Depletion of FGFR4 inhibits AKT and STAT3 phosphorylation

**Figure 3:**
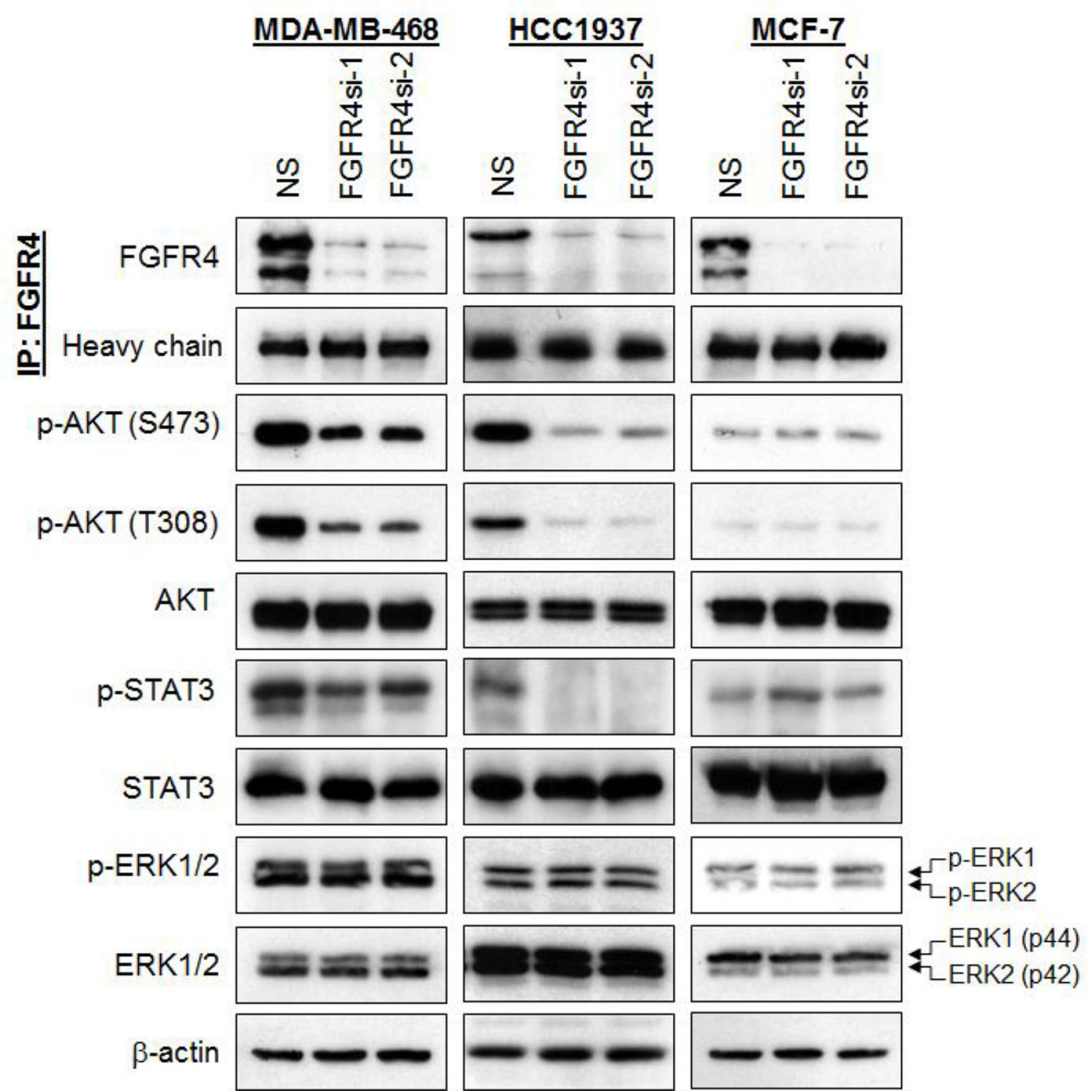
Depletion of endogenous FGFR4 reduces AKT and STAT3 phosphorylation FGFR4 depletion downregulates AKT and STAT3 phosphorylation in basal-like MDA-MB-468 and HCC1937 but not in MCF-7 cells. Lysates were harvested and assessed by IP-Western analysis 96 h post-transduction. Heavy chain and β-actin serve as loading controls. Note the downregulation of phospho-AKT and phospho-STAT3.

FGFR family members have been shown to mediate their biological activities through several signaling pathways, including activation of AKT, STAT3 and ERK1/2 [13]. To evaluate whether the pro-survival effects of FGFR4 in breast cancers cells could be mediated through aberrant activation of these pathways, we analyzed the effects of FGFR4 knockdown on these targets using a panel of phospho-specific antibodies. As shown in Figure 3, depletion of endogenous FGFR4 in MDA-MB-468 and HCC1937 reduced AKT phosphorylations at serine 473 (S473) and threonine 308 (T308). Interestingly, STAT3 phosphorylation was also reduced in HCC1937, and to a lesser extent in MDA-MB-468 following FGFR4 depletion, while the phosphorylation of ERK1/2 remained unchanged. No such changes were observed in MCF-7 cells, which are insensitive to FGFR4 depletion.

### FGFR4 mediates the survival of MDA-MB-468 and HCC1937 cells via AKT signaling pathway

**Figure 4:**
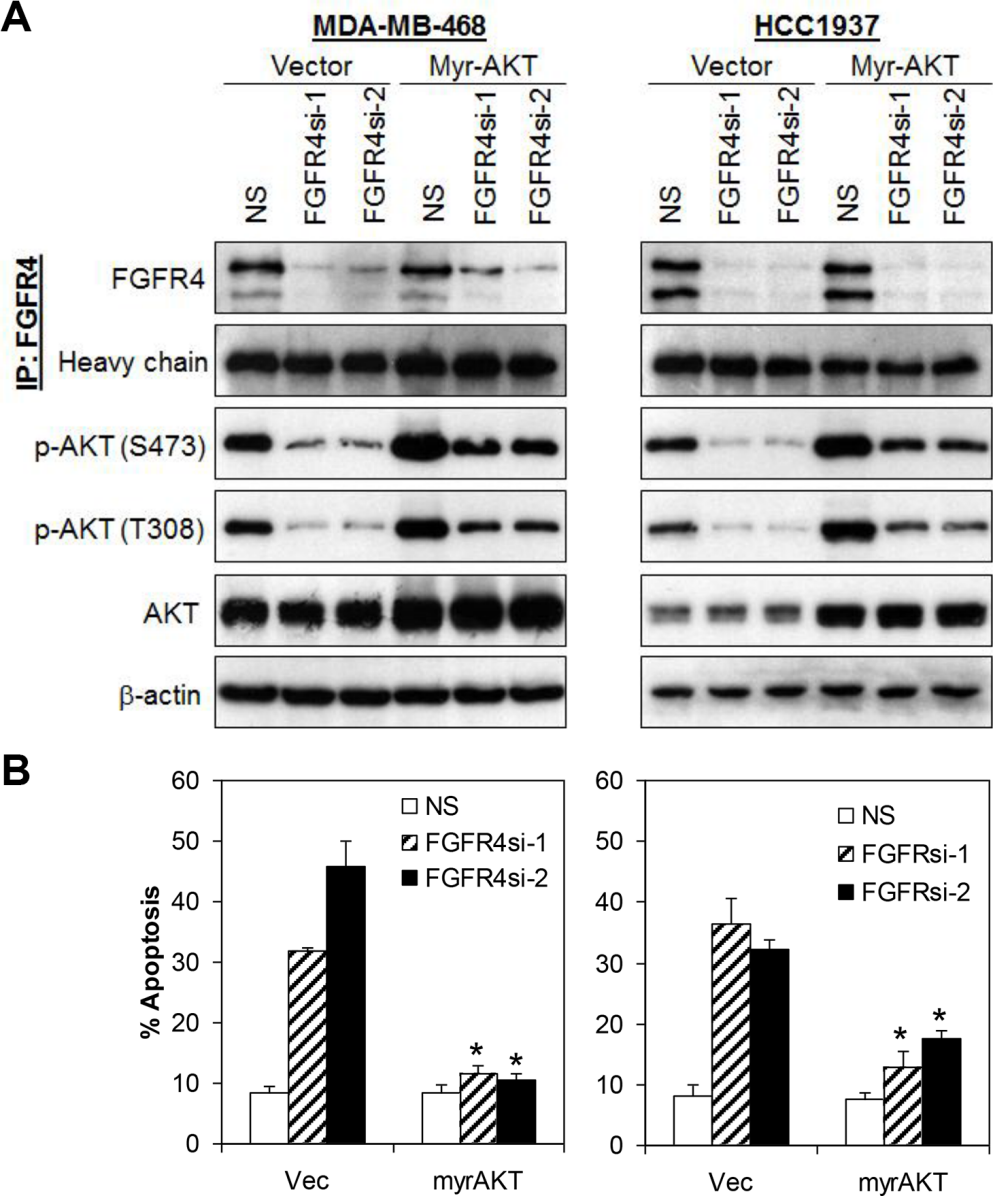
FGFR4 mediates cells survival *via* activation of AKT signaling pathway (**A**) Ectopic expression of constitutively active AKT (Myr-AKT) rescued apoptotic cell death induced by FGFR4 knockdown. Cells were transiently transfected with myristoylated AKT concurrently with either NS or FGFR4 targeting shRNAs. Lysates were collected 72 h post-transfection and analyzed by IP-Western blotting. (**B**) Ectopic expression of myristoylated AKT abrogated FGFR4-depletion induced cell death in basal-like MDA-MB-468 and HCC1937 cells. Cells were transfected as in (A). Apoptosis was analyzed by annexin V/7-AAD staining. Bars represent means ± s.d. of three independent experiments. (*) indicates statistical significance compared with vector control cells following FGFR4 depletion (*P* < 0.01, Student's *t*-test).

Since FGFR4 knockdown in MDA-MB-468 and HCC1937 decreases AKT phosphorylation, and AKT is known to play an essential role in cell survival [38, 39], we postulate that AKT hyperactivation may sustain the survival of these breast cancer cells. To test this hypothesis, MDA-MB-468 and HCC1937 cells were co-transfected with a constitutively active myristoylated AKT (Myr-AKT) and FGFR4 shRNA followed by evaluation of apoptotic cell death using Annexin V/7-AAD flow cytometry. As shown in Figure 4 and Supplementary Figure S2, ectopic expression of Myr-AKT completely abrogated the apoptotic cell death induced by FGFR4 depletion. In contrast, no such effect was observed in cells overexpressing a constitutively active STAT3 (data not shown), suggesting that FGFR4 mediates the survival of MDA-MB-468 and HCC1937 cells mainly through the AKT signaling pathway.

### FGF19 is secreted in a subset of basal-like breast cancer cells

**Figure 5:**
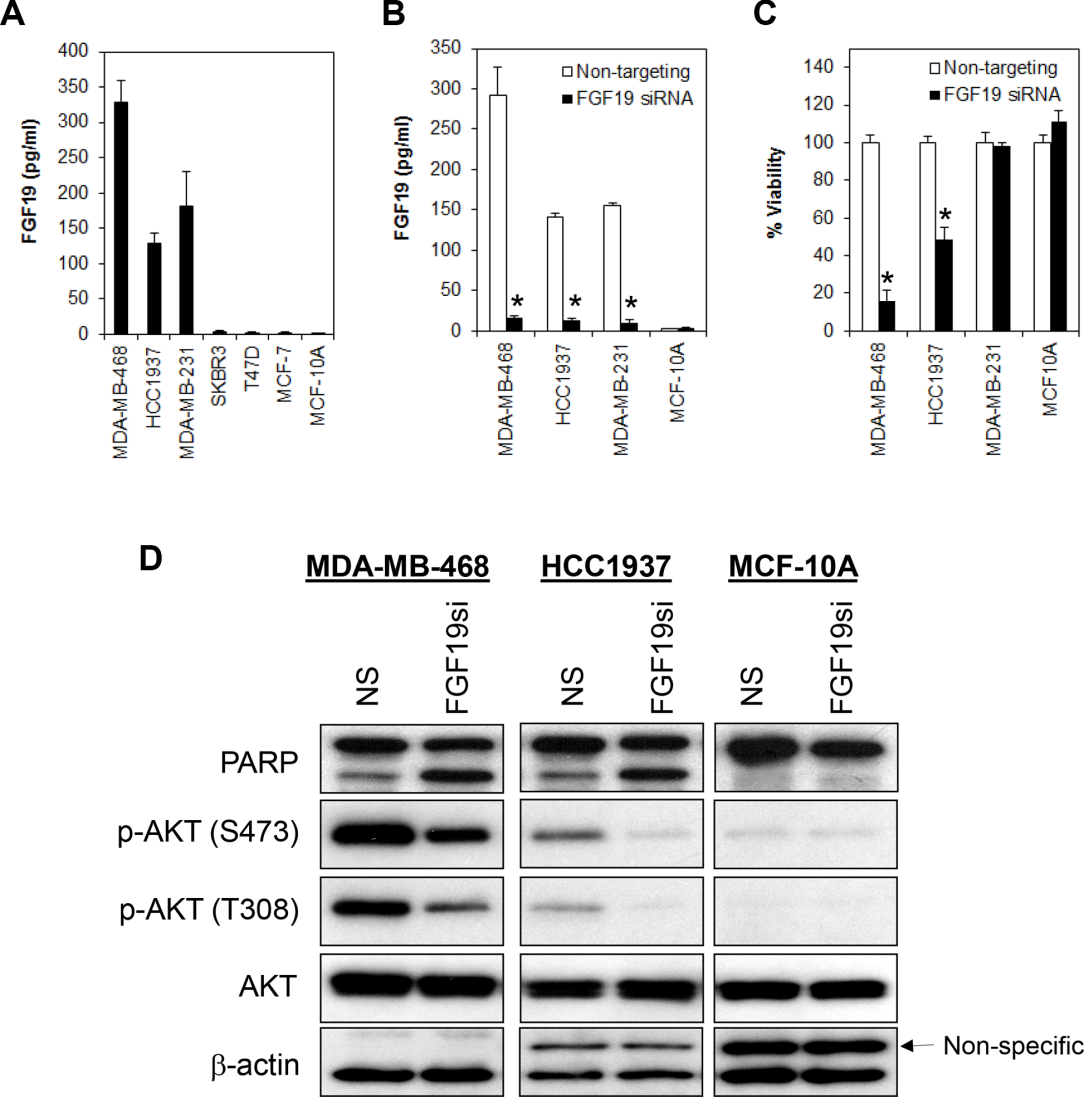
FGF19 autocrine signaling promotes cell survival in FGFR4/FGF19 co-expressed cells (**A**) FGF19 is secreted in a subset of breast cancer cells. Supernatant was collected from all pre-confluent cells following 72 h of seeding and FGF19 secretion was measured using ELISA immunoassay. Note that no FGF19 was detected in the luminal-like (MCF-7, T47D and SKBR3) breast cancer cells nor in non-transformed myoepithelial cells (MCF-10A). (**B** and **C**) Depletion of FGF19 induces growth inhibition in the FGFR4^+^/FGF19^+^ MDA-MB-468 and HCC1937 but not in the FGFR4^−^/FGF19^+^ MDA-MB-231 or in the FGFR4^−^/FGF19^−^ MCF-10A non-transformed myoepithelial cells. Cells were transfected with a FGF19 smart pool siRNA as in (A) and the cell viability measured by CellTiter-Glo assay 3 days post-transfection. Bars represent means ± s.d. of three independent experiments. (*) indicates statistical significance compared with control cells transfected with a non-targeting siRNA (*P* < 0.01, Student's *t*-test). (**D**) Depletion of FGF19 downregulates AKT phosphorylation.

Next, we sought to investigate the mechanism by which FGFR4 might be constitutively activated in a subset of breast cancer cells. Studies have shown that FGFR4 signaling activation could be triggered by various FGFs, particularly FGF19, which has a unique receptor high-affinity binding specificity towards FGFR4 [40]. We hypothesized that the constitutive activation of FGFR4 in MDA-MB-468 and HCC1937 could be mediated by an autocrine secretion of FGF19 as has been shown in other cancers, such as hepatocellular carcinomas, lung squamous cell carcinomas, and colon adenocarcinomas [41–43]. Indeed, using a highly sensitive and specific FGF19 ELISA assay, we show that the basal-like MDA-MB-468, MDA-MB-231 and HCC1937 secrete a substantial amount of FGF19 (approx. 100–350 pg/mL) (Figure 5A). In contrast, no FGF19 was detected in SKBR3, T47D, MCF7 and MCF-10A cells.

**Figure 6:**
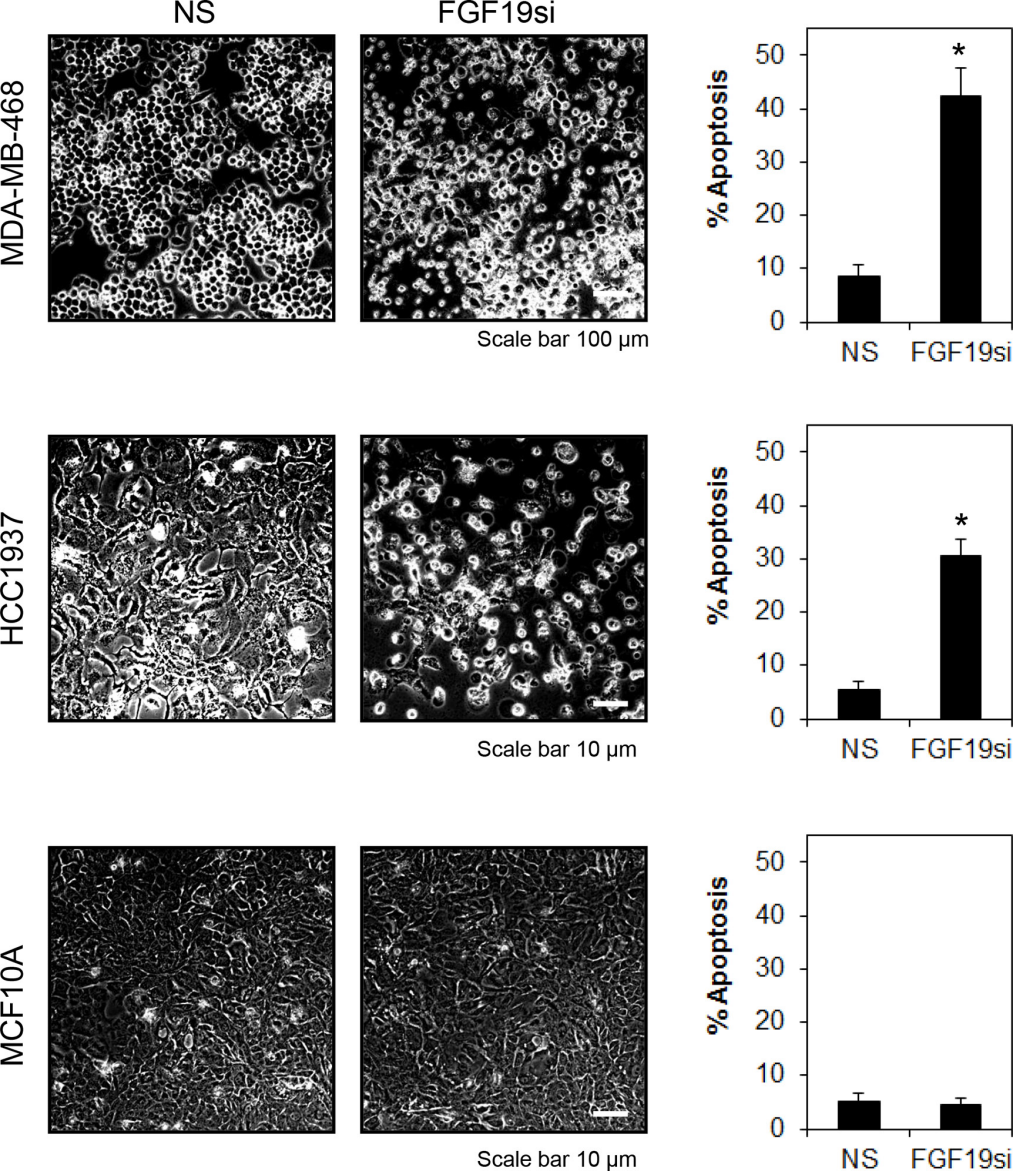
FGFR4-FGF19 autocrine signaling promotes survival in MDA-MB-468 and HCC1937 Cells were treated as in Figure 5. Morphological changes were recorded at 100X magnification, 72 h post-transfection. Apoptosis was determined by annexin V/7-AAD staining. Bars represent means ± s.d. of three independent experiments. (*) indicates statistical significance compared with non-targeting siRNA (*P* < 0.01, Student's *t*-test).

Since FGF19 is secreted in both MDA-MB-468 and HCC1937, and these cells are susceptible to FGFR4-knockdown induced apoptosis, it is likely that the constitutive activation of FGFR4 is mediated by autocrine FGF19. To test this hypothesis, we transiently depleted FGF19 from MDA-MB-468, HCC1937, MDA-MB-231 and MCF-10A cells using a FGF19-specific siRNAs pool. Similar to the depletion of FGFR4, depletion of endogenous FGF19 in MDA-MB-468 and HCC1937 cells also significantly reduced cell viability, an observation that is corroborated by the induction of poly ADP ribose polymerase (PARP) cleavage and reduction in AKT phosphorylation (Figure 5B–5D). Consistently, we also observed significant apoptotic cell death as indicated by morphological changes and annexin V/7-AAD staining (Figure 6). No such effect was observed in the FGFR4^−^/FGF19^+^ MDA-MB-231 cells or in the FGFR^−^/FGF19^−^ MCF-10A cells, suggesting that only cancer cells that co-expressed FGFR4 and FGF19 might be susceptible to FGFR4/FGF19 inhibition.

### A neutralizing anti-FGF19 monoclonal antibody blocks cancer cell growth

**Figure 7:**
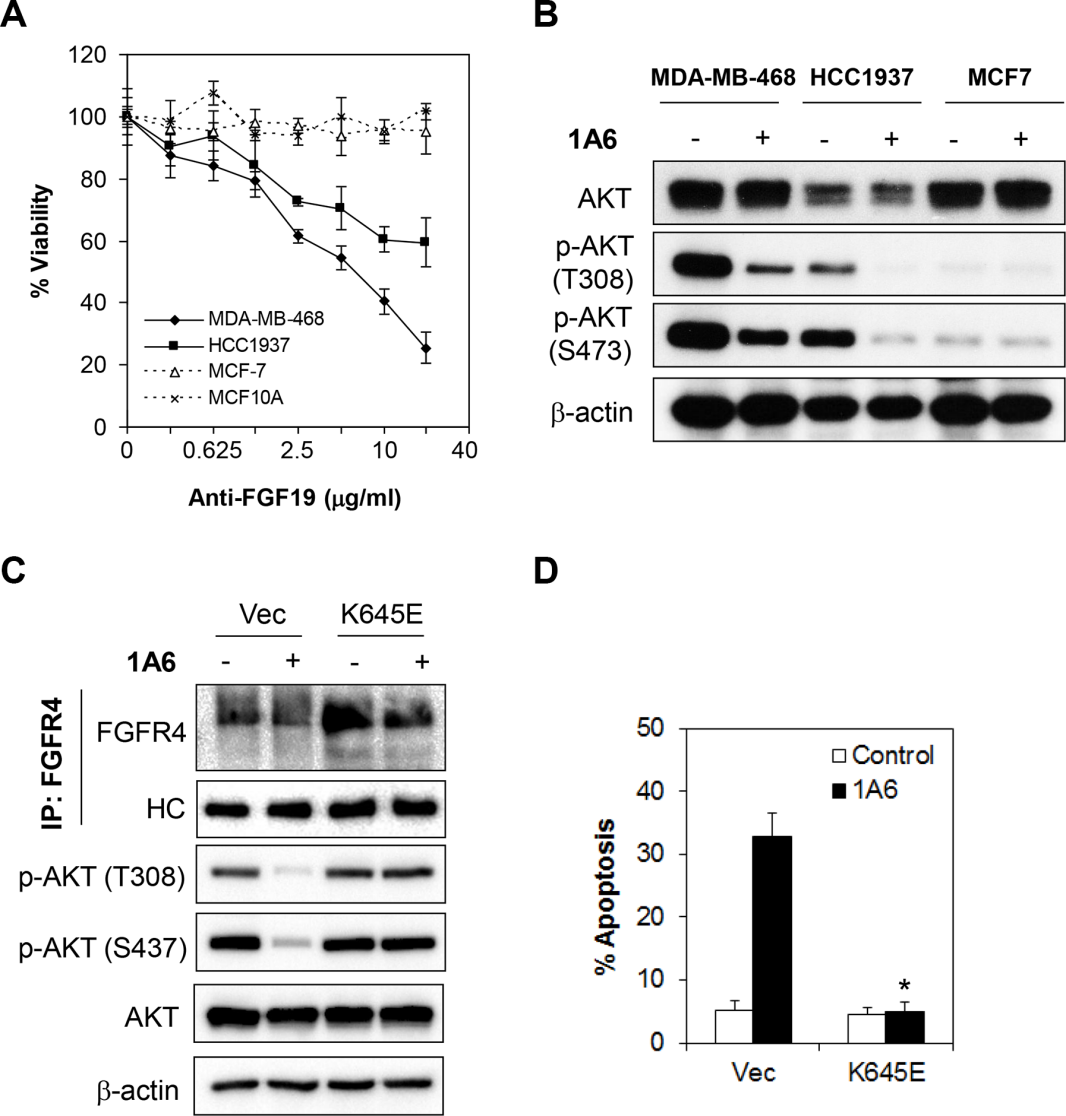
Inactivation of autocrine FGF19 by monoclonal antibody abrogates FGFR4-mediated survival of breast cancer cells (**A**) Neutralization of FGF19 by an anti-FGF19 antibody, 1A6, inhibits cell growth in FGFR4^+^/FGF19^*+*^ MDA-MB-468 and HCC1937 cells, but not in FGFR4^+^/FGF19^−^ MCF-7 cells or in FGFR4^−^/FGF19^−^ MCF-10A cells. Cells were treated with various concentrations of 1A6 for 72 h and the cell viability was determined by CellTiter-Glo assay. (**B**) 1A6 attenuates AKT phosphorylation. Cells were treated with 10 μg/mL of 1A6 for 48 h and lysates were collected for Western blot analyses. (**C** and **D**) The apoptotic effect of 1A6 is dependent on inhibition of FGFR4/FGF19 signaling. Cells were transfected with vector or constitutively active FGFR4 K645E mutant for 24 h followed by treatment with 10 μg/mL of 1A6 for 72 h. Apoptosis was analyzed by annexin V/7-AAD staining. Bars represent means ± s.d. of three independent experiments. (*) indicates statistical significance compared with vector control cells following 1A6 treatment (*P* < 0.01, Student's *t*-test).

We next sought to test the potential benefit of targeting FGF19 therapeutically in breast cancer cells that co-express FGFR4 and FGF19. We assayed the effect of neutralizing FGF19 on the cancer cell proliferation of MDA-MB-468 and HCC1937 cells using a previously characterized neutralizing antibody specific against FGF19 (1A6) [41]. Cells were treated with 1A6 antibody for 72 h and the cell viability measured by MTT cell proliferation assay. As shown in Figure 7A the anti-FGF19 antibody had a dramatic inhibitory effect on cell proliferation in the FGFR4^+^/FGF19^+^ MDA-MB-468 and HCC1937 cells. However, no such inhibitory effect was observed in MCF10A cells, which are FGFR4^−^/FGF19^−^ or in MCF-7 cells, which are FGFR4^+^/FGF19^−^. Furthermore, 1A6 treatment also diminishes the phosphorylation of AKT in both MDA-MB-468 and HCC1937 but not in MCF7, which is consistent with the results observed following FGF19 knockdown by siRNA (Figure 7B).

To further validate whether the effect of 1A6 anti-FGF19 antibody was indeed mediated through the FGFR4/FGF19 autocrine axis, we overexpressed a constitutively active FGFR4 K654E mutant in MDA-MB-468 and HCC1937 cells, followed by quantitation of 1A6-induced apoptosis [44, 45] (Figure 7C). Indeed, the ectopic expression of the constitutively active FGFR4 K645E mutant completely abrogated the apoptotic effects induced by 1A6 (Figure 7D and Supplementary Figure S3), suggesting that the effects of 1A6 is dependent on FGFR4/FGF19 signaling, which is consistent with previous studies [41, 43, 46].

### Inhibition of FGFR4/FGF19 autocrine axis enhances doxorubicin sensitivity in breast cancer cells

**Figure 8:**
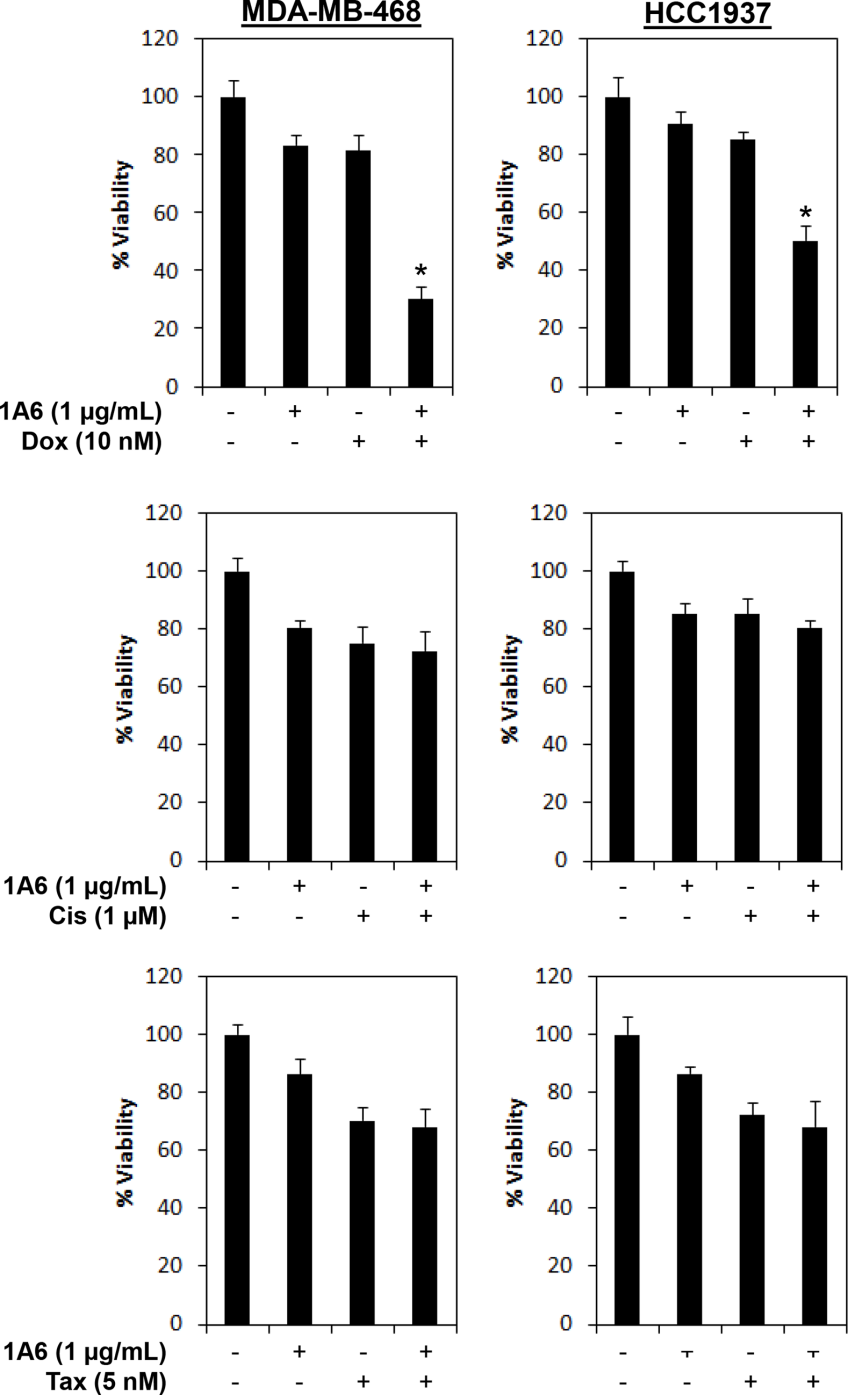
Inhibition of FGF19 synergizes doxorubicin sensitivity in FGFR4/FGF19 co-expressed breast cancer cells Cells were treated with 1 μg/mL of 1A6 and/or 10 nM of doxorubicin (Dox), 1 μM of cisplatin (Cis) or 5nM of Paclitaxel (Tax). Cell viability was determined by CellTiter-Glo assay 72 h following treatments. (*) indicates statistical significance compared with 1A6 or doxorubicin treated cells (*P* < 0.01, Student's *t*-test).

Since FGFR4 upregulation and activation has been recently shown to confer chemoresistance in breast cancer cells [47], we asked whether inhibition of the FGFR4/FGF19 axis might enhance chemotherapy sensitivity in cancer cells. To test this hypothesis, we evaluated the effects of the combined application of 1A6 with doxorubicin, cisplatin or paclitaxel in the FGFR4^+^/FGF19^+^ MDA-MB-468 and HCC1937 cells. As shown in Figure 8, inhibition of FGFR4/FGF19 autocrine axis enhances doxorubicin, but not cisplatin or paclitaxel, sensitivity in MDA-MB-468 and HCC1937 breast cancer cells, suggesting that the anti-FGF19 monoclonal antibody might potentiate sensitivity of refractory tumor cells to chemotherapy.

### FGFR4 and FGF19 are co-expressed in a subset of primary breast tumors

**Figure 9:**
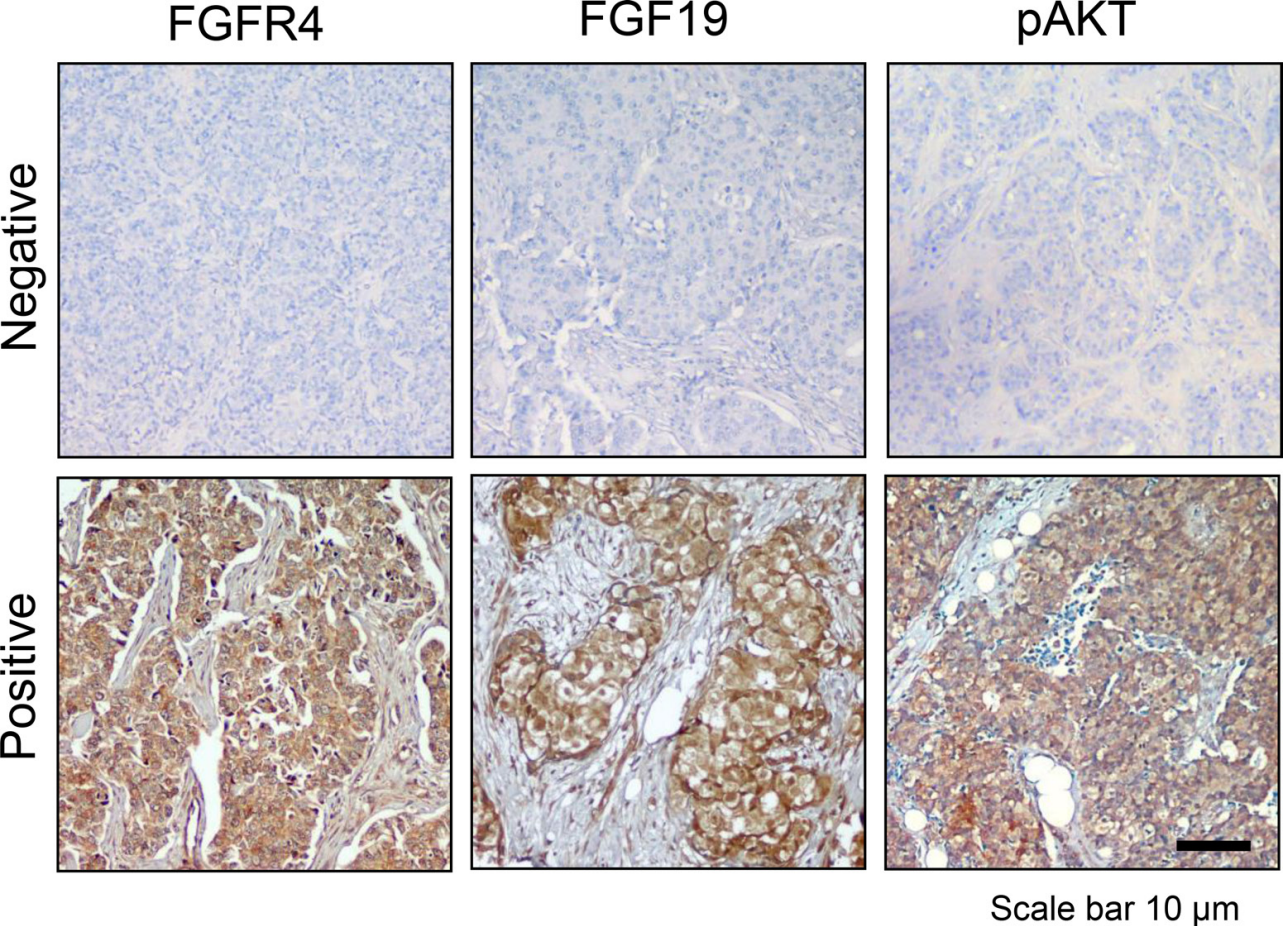
FGFR4/FGF19 co-expression is associated with AKT phosphorylation in a subset of breast cancer cells Immunohistochemistry of representative primary tumors. Photomicrographs demonstrate low and high expression of FGFR4, FGF19 and phospho-AKT (S473). Note the positive staining for FGFR4, FGF19 and phospho-AKT in the cytoplasm of most tumor cells, but not in the nucleus. The association of FGFR4/FGF19 expression with clinicopathological features are presented in Tables 2 and 3. Original magnification, 100X.

Finally, to determine whether FGFR4/FGF19 co-expression is present in primary breast tumors, we stained tissue microarrays of primary human breast cancer samples diagnosed with invasive ductal carcinoma (IDC) for FGFR4 and FGF19, and compared this with AKT phosphorylation (Figure 9). As shown in Table 2, approximately 26.5% of the tumors were FGFR4^−^/FGF19^−^, 45% of the tumors were positive for either FGFR4 or FGF19, and 28.6% of the primary IDC exhibit co-expression of FGFR4 and FGF19. Importantly, FGFR4/FGF19 co-expression was associated with AKT phosphorylation (*P* < 0.001), Ki-67 staining (*P* = 0.005) and higher tumor stage (*P* < 0.001) (Table 3). Interestingly, FGFR4/FGF19 co-expression was also strongly associated with basal-like phenotype, with up to 43.6% of the triple negative (ER/PR/HER2 negative) tumors and 55.9% of the CK5/6 positive tumors exhibiting FGFR4/FGF19 co-expression. In contrast, no significant association between FGFR4/FGF19 co-expression and EGFR or p53 was observed, indicating that the FGFR4/FGF19 axis is independent of EGFR or p53 signaling.

**Table 2:** Expression of FGFR4 and FGF19 in primary breast tumors

	FGFR4–	FGFR4+	*P*-value^ψ^
FGF19–	76 (26.5%)	43 (15.0%)	0.033^*^
FGF19+	86 (30.0%)	82 (28.6%)	

ψ Chi-square test;

* *P* < 0.05.

**Table 3:** Association of FGFR4/FGF19 co-expression with clinicopathological features of invasive breast cancers

	FGFR4/FGF19 Co-expression				Total (*n* = 287)		*P*-Value^Ψ^
	No (*n* = 205)		Yes (*n* = 82)				
**TNM stage**							
I	5	(1.7%)	4	(1.4%)	9	(3.1%)	< 0.001^*^
II	182	(63.4%)	23	(8.0%)	205	(71.4%)	
III	12	(4.2%)	38	(13.2%)	50	(17.4%)	
IV	6	(2.1%)	17	(5.9%)	23	(8.0%)	
**ER/PR/HER2 status**							
ER/PR–, HER2–	53	(18.5%)	41	(14.3%)	94	(32.8%)	< 0.001^*^^#^
ER/PR–, HER2+	33	(11.5%)	13	(4.5%)	46	(16.0%)	
ER/PR+, HER2–	96	(33.4%)	20	(7.0%)	116	(40.4%)	
ER/PR+, HER2+	23	(8.0%)	8	(2.8%)	31	(10.8%)	
**CK5/6**							
Negative	164	(57.1%)	30	(10.5%)	194	(67.6%)	< 0.001^*^
Positive	41	(14.3%)	52	(18.1%)	93	(32.4%)	
**pAKT**							
Negative	180	(62.7%)	52	(18.1%)	232	(80.8%)	< 0.001^*^
Positive	25	(8.7%)	30	(10.5%)	55	(19.2%)	
**Ki-67**							
Low	114	(39.7%)	31	(10.8%)	145	(50.5%)	0.005^*^
High	89	(31.0%)	51	(17.8%)	140	(48.8%)	
Unknown	2	(0.7%)	0	(0.0%)	2	(0.7%)	
**p53**							
Negative	81	(28.2%)	37	(12.9%)	118	(41.1%)	0.826
Positive	93	(32.4%)	40	(13.9%)	133	(46.3%)	
Unknown	31	(10.8%)	5	(1.7%)	36	(12.5%)	
**EGFR**							
Negative	142	(49.5%)	58	(20.2%)	200	(69.7%)	0.967
Positive	57	(19.9%)	23	(8.0%)	80	(27.9%)	
Unknown	6	(2.1%)	1	(0.4%)	7	(2.4%)	

ψ Chi-square test;

* *P* < 0.05;

# Statistical significance between triple-negative (ER/PR–, HER2–) vs non-triple negative (ER/PR–, HER2+; ER/PR+, HER2–; and ER/PR+, HER2+) breast cancers (*P* < 0.01).

Collectively, our results demonstrated the existence of a FGFR4-FGF19 autocrine loop, which could potentially be developed as a therapeutic target for future treatment of refractory basal-like breast cancers.

## DISCUSSION

The significance of FGFs/FGFRs signaling deregulation in breast cancers has been documented in several studies [13, 48, 49]. However, the exact mechanism by which each FGFR family protein might mediate the survival and proliferation of cancer cells remained unknown. Through an unbiased lentiviral shRNA kinome library screen, we identified FGFR4 as a receptor tyrosine kinase that is required for the survival of a subset of basal-like breast cancer cells. We found that FGFR4 is overexpressed in a subset of breast cancer cell lines but not in the normal myoepithelial cells. Of note, the FGFR4 protein was found to be phosphorylated in breast cancer cells that express it, suggesting that FGFR4 might be constitutively active in these cancer cells. These results are consistent with previous studies, which show that FGFR4 is overexpressed in 10–30% of breast cancers [50–52].

Unlike FGFR1-3, where activating mutations and genetic amplifications are commonly associated with tumor progression, FGFR4 is rarely mutated in human cancers [13, 53, 54]. Most of the reports regarding FGFR4 and cancers have focused mainly on a common single-nucleotide polymorphism (SNP), FGFR4 Gly388Arg, of which at least one copy is present in approximately 30– 50% of the population [13, 55]. This SNP does not seem to increase the incidence of cancer, but has been reported to be associated with poor prognosis in multiple cancer types, including breast cancer, and is thought to promote cancer cell motility and invasion or resistance to chemotherapy [20, 47, 56–61]. However, sequence analysis of breast cancer cell lines did not show any correlation between the Gly388 SNP genotype with FGFR4 mRNA or protein expression levels, suggesting that FGFR4 overexpression might have a different functional role in the pathogenesis of breast cancer (Supplementary Table S1).

To test whether FGFR4 plays a functional role in mediating the survival of basal-like breast cancer cells, gene knockdown was performed using two independent lentiviral shRNAs targeting the endogenous FGFR4 in a panel of breast cell lines. Indeed, depletion of the endogenous FGFR4 in the basal-like MDA-MB-468 and HCC1937 induces a significant amount of growth inhibition and apoptosis, while no such effects were observed in MDA-MB-231, T47D, MCF-10A and HMEC cells, which do not express FGFR4. Intriguingly, depletion of endogenous FGFR4 has no such effect on the luminal-like MCF-7 and HER2-amplified SKBR3 cells, suggesting that FGFR4 mediates cancer cell survival in a cell context-dependent manner.

Activation of FGFRs has been shown to activate AKT, ERK1/2 and STAT3 signaling [13, 62]. Activated FGFRs phosphorylates FRS2 on several sites, allowing the recruitment of the adaptor proteins son of sevenless (SoS) and growth factor receptor bound 2 (GRb2) to activate RAS and the downstream RAF and MAPK pathways. A separate complex involving GRb2-associated-binding protein 1 (GAb1) recruits a complex, which includes PI3K, and this activates an AKT-dependent anti-apoptotic pathway [63]. Activation of FGFRs also phosphorylates and activates STAT3 directly, independent of FRS2 [45].

To test whether the pro-survival effects of FGFR4 are mediated by MAPK, AKT and/or STAT3 pathways, we investigated the effects of FGFR4 depletion on these pathways. Our results demonstrated that depletion of FGFR4 reduced AKT and STAT3 phosphorylation while the phosphorylation of ERK1/2 remained unchanged. Importantly, ectopic expression of a myristoylated AKT, but not the constitutively active STAT3, significantly abrogated apoptosis induced by FGFR4 knockdown, suggesting that the pro-survival effects of FGFR4 are mediated mainly through the PI3K/AKT signaling. These results are consistent with a recent study that shows that inhibition of the FGFR activity by TKI258 significantly inhibits AKT activity and AKT-dependent cell proliferation in murine breast cancer cells [64].

Since FGFR4 is rarely mutated and the G388 polymorphism is not associated with the pro-survival activities of FGFR4, we asked whether the constitutively active FGFR4 signaling in cancers could be activated by an autocrine signaling loop, as has been demonstrated in other FGFR family members [65]. To test this hypothesis, the protein expression of a highly specific ligand of FGFR4, FGF19, was evaluated by ELISA. Our results show that the basal-like breast cancer cell lines express high levels of secreted FGF19 as compared to luminal-like breast cancers or non-transformed mammary myoepithelial cells. Phenotypic and cell viability assessment following depletion of FGF19 also demonstrated that FGF19-mediated downstream signaling is crucial for the survival of basal-like breast cancer cells that co-express FGFR4 and FGF19 (MDA-MB-468 and HCC1937), as depletion of FGF19 by siRNA or anti-FGF19 monoclonal antibody (1A6) inhibits cell proliferation and induces apoptosis in these cells. In stark contrast, no such effects were observed in the FGFR4^+^/FGF19^−^ MCF-7 cells or in the FGFR4^−^/FGF19^+^ MDA-MB-231 cells. Of note, the mechanism by which FGFR4 is constitutively active in the absence of FGF19 in MCF-7 and SKBR3 cells remains to be further investigated. It is likely that aberrant expression of other FGFs, such as FGF1 and FGF3, which have been shown to bind FGFR4 at high concentration [66], might regulate the phosphorylation of FGFR4 in these cells. Regardless, the knockdown of FGFR4 has no effect on MCF-7 and SKBR3 cells, suggesting that FGFR4 signaling is dispensable in mediating the survival of these cells.

Since hyperactivation of FGFR4 signaling has also been shown to confer chemoresistance in breast, gastric and colorectal cancer cells [47, 67–70], we hypothesized that the combined application of anti-FGF19 neutralizing antibody and chemotherapeutic agents might further increase the apoptosis rate of cancer cells. Indeed, inhibition of FGFR4/FGF19 autocrine signaling synergizes with doxorubicin (but not cisplatin or paclitaxel) to enhance sensitivity in basal-like breast cancer cells, highlighting the potential of using an anti-FGF19 monoclonal antibody as a therapeutic for breast cancer cells that co-express FGFR4 and FGF19. Whether the specific synergism between FGFR4/FGF19 inhibition and doxorubicin is dependent on PI3K/AKT signaling remains to be further investigated.

Finally, using a panel of well-validated tissue microarray, we show that a subset of primary breast tumors co-expresses FGFR4 and FGF19. Importantly, the co-expression of FGFR4/FGF19 is significantly associated with AKT phosphorylation, Ki-67 staining, higher tumor stage and basal-like phenotype (triple-negative and CK5/6 positivity) in breast cancers. In contrast, no association between FGFR4/FGF19 co-expression and EGFR or p53 was observed, suggesting that FGFR4/FGF19 might mediate cancer cell survival through a mechanism independent of EGFR or p53 signaling.

In summary, our results demonstrate that the FGFR4/FGF19 autocrine signaling mediates the survival of a subset of basal-like breast cancer cells through activation of PI3K/AKT signaling. Inhibition of the FGFR4/FGF19 signaling induces tumor-specific cell death in cancer cells that express them. As such, targeting the FGFR4/FGF19 autocrine loop represents a potential therapeutic strategy for future management of refractory basal-like breast cancers.

## MATERIALS AND METHODS

### Materials

Constitutively active myristoylated AKT (Addgene plasmid # 9008), STAT3 (Addgene plasmid # 24983) were a gift from William Sellers [71] and Linzhao Cheng [72], respectively. FGF19 antibody (clone 1A6) was provided by Genentech Inc., USA. The constitutively active FGFR4-K645E has been described previously [45].

### Cell lines and cell culture

The human breast cancer cell lines MDA-MB-468, MDA-MB-231, HCC1937, SKBR3, T47D and MCF7 were purchased from American Type Culture Collection (ATCC) and maintained in RPMI-1640 medium containing 10% fetal bovine serum, 100 IU/ml penicillin, and 100 μg/ml streptomycin (Sigma-Aldrich, St. Louis, MO, USA). The non-transformed human mammary epithelial cells MCF10A were cultured in DMEM-F12 (Invitrogen Carlsbad, CA) supplemented with 5% horse serum, 20 ng/ml EGF, 0.5 μg/ml hydrocortisone, 10 μg/ml insulin, 100 IU/ml penicillin, and 100 μg/ml streptomycin, while HMEC was grown in HuMEC Ready Media (Invitrogen, Carlsbad, CA). All cells were maintained at 37°C in 5% CO_2_. Cells were passaged for less than 6 months and no further authentication was performed by the authors.

### Lentiviral human kinase shRNA library screen

The role of protein kinases was examined using the MISSION LentiExpress™ Human Kinases shRNA Library (Sigma, St Louis, MO). Briefly, MDA-MB-468 basal-like breast cancer cells were seeded at a density of 1500 cells/well in a 96-well plate overnight. Cells were transduced with lentiviral particles at multiplicities of infection (MOI) of approximately 1.5 in the presence of 7.5 μg/ml polybrene (Sigma, St Louis, MO) for 18 h in 37°C, 5% CO_2_. Medium was changed and the number of viable cells was determined using CellTiterGlo assay (Promega, Madison, WI) 5 days post-transduction. Controls include lentiviral particles carrying an empty vector (pLKO.1-puro), a non-target shRNA (NS) or a GFP-expressing lentiviral construct to monitor transduction efficiency and well-to-well difference. All data were normalized against non-target shRNA controls. Hits were identified based on: 1) at least two independent shRNAs targeting a specific gene exhibit a *Z*-score of less than −2; and 2) the *P*-value of Redundant siRNA Activity (RSA) Analysis of each kinase is less than 0.05 [73].

### Quantitative real-time PCR (qPCR) analysis

Gene expression levels were measured by real-time qPCR as described previously [74, 75]. The specific forward and reverse primer sequences are shown in Supplementary Table S2. The conditions for all qPCR reactions were as follows: 3 min at 94°C followed by 40 s at 94°C, 40 s at 60°C, and 25 s at 72°C for 40 cycles. The expression data were normalized to *GAPDH* as a house-keeping gene.

### Protein isolation, immunoprecipitation (IP) and Western blot analysis

Protein lysates from cells were extracted in ice-cold lysis buffer consisting of 1% NP-40, 1 mM DTT, protease inhibitors (Roche, Indianapolis, USA) and phosphatase inhibitor I and II cocktails (Sigma-Aldrich, St. Louis, MO, USA) in PBS. For Western blot analysis, total protein (50 μg) was subjected to SDS-PAGE followed by immunoblotting. For IP, precleared lysates (500 μg) were rocked with 2 μg of anti-FGFR4 polyclonal antibody (C-16; Santa Cruz Biotechnology, CA, USA) for 2 h at 4°C. The immunocomplexes were precipitated with protein A sepharose (GE Healthcare Bio-Sciences AB, Uppsala, Sweden) and washed three times with lysis buffer prior to SDS-PAGE and immunoblotting analyses. Monoclonal antibodies against FGFR4 and β-actin were purchased from Santa Cruz Biotechnology (CA, USA). Primary antibodies against AKT, p-AKT (Ser473), p-AKT (Thr308), ERK1/2, p-ERK1/2, STAT3, p-STAT3 (Tyr705), PLCγ, p-PLCγ (Tyr783) and PARP were obtained from Cell Signaling Technology (Danvers, MA, USA). Monoclonal antibody against phosphotyrosine (4G10) was purchased from Millipore Corporation (Bedford, MA, USA).

### Lentiviral production and transduction

Lentiviral non-targeting shRNA (NS) and shRNA constructs targeting FGFR4 were purchased from Sigma-Aldrich (St. Louis, MO, USA). The shRNAs target sequences are presented in Supplementary Table S3. Lentiviral particles were generated by co-transfection of respective shRNA constructs with lentiviral packaging plasmids, psPAX2 (Addgene plasmid #1 2260) and envelope plasmids, pMD2.G (Addgene plasmid # 12259) into HEK-293T cells, as described previously [76–78].

### Transfection

Cells were seeded overnight in 96-well plates at a density of 5.0 × 10^3^ cells/well prior to transfection with either of two pools of siRNAs – ON-TARGETplus Non-targeting Control Pool siRNAs and ON-TARGETplus FGF19 siRNAs (Thermo Scientific Dharmacon, Lafayette, CO, USA) using X-tremeGENE siRNA Transfection Reagent (Roche Diagnostics, IN, USA).

### Detection of apoptosis by annexin V flow cytometry

Apoptosis was quantified using a PE Annexin V Apoptosis Detection Kit (BD Biosciences, USA) according to the manufacturer's instructions. The cells were analyzed using a FACSCalibur flow cytometer and the CellQuest Pro software (version 5.1.1; BD Biosciences, USA).

### FGF19 enzyme-linked immunosorbent assay (ELISA)

Cells were seeded in 6-well plates at a density of 5 × 10^5^ cells/well and supernatants were collected after 72 h. The level of FGF19 was determined using FGF19 Quantikine ELISA kit (R&D Systems, Mineapolis, USA) according to the manufacturer's protocol and analyzed by Tecan infinite F200 microplate reader (Männedorf, Switzerland) at an absorbance wavelength of 450 nm and a correction wavelength of 540 nm or 570 nm.

### Tissue microarrays

Formalin-fixed, paraffin-embedded breast cancer tissue microarray (Pantomics, Richmond, CA) containing 287 nonoverlapping tissue samples were deparaffinized and rehydrated. Heat-induced epitope retrieval (HIER) method was performed to retrieve all the antigenic determinants. Primary antibodies against FGFR4 (Clone C-16) and FGF19 (MAB969) were purchased from Santa Cruz Biotechnology (CA, USA) and R&D Systems (Mineapolis, USA), respectively. Antibodies against phospho-AKT-S473 (Clone 14–5), p53 (Clone DO-7), EGFR (clone E30), CK5/6 (clone D5/16 B4), and Ki-67 (clone MIB-1) were supplied by Dako Corporation (Carpinteria, CA, USA). Positivity was defined as the presence of 1% or more positively stained tumor cells as described previously [79–82].

### Statistical analysis

The data were analyzed using SPSS version 17.0 (SPSS, INC, Chicago, IL). The chi-square test was used to evaluate any potential association between the FGFR4/FGF19 co-expression and the clinicopathologic parameters. A *P*-value < 0.05 was considered statistically significant.

## SUPPLEMENTARY MATERIALS FIGURES AND TABLES


