# Supplementary material for: Fibroblast growth factor receptor 4 (FGFR4) and fibroblast growth factor 19 (FGF19) autocrine enhance breast cancer cells survival
Source: Oncotarget. 2016 May 12;7(36):57633–50. doi: 10.18632/oncotarget.9328 (PMC5295378; doi:10.18632/oncotarget.9328)
Supplement: Supplementary file 1 [file oncotarget-07-57633-s001.pdf]

Fibroblast growth factor receptor 4 (FGFR4) and fibroblast growth factor 19 (FGF19) autocrine enhance breast cancer cells survival

Supplementary Materials

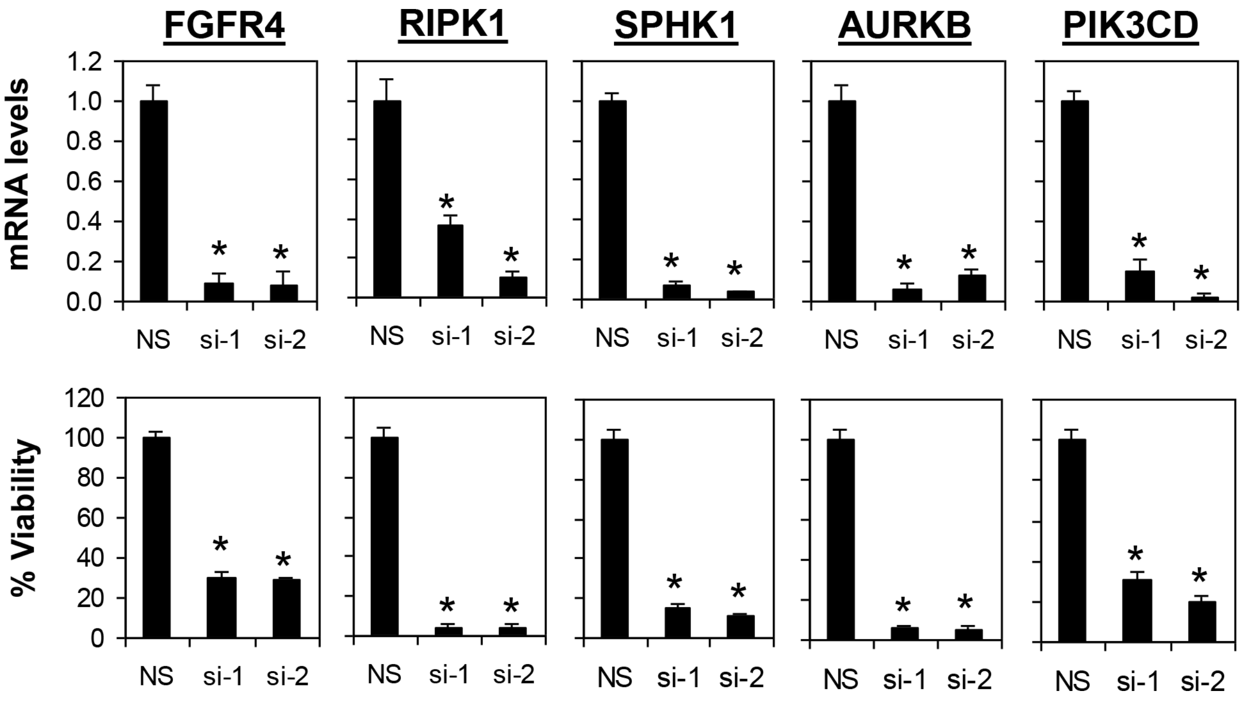

Supplementary Figure S1: Validation of hits identified from the primary screen. The levels of gene knockdown were evaluated using 2 independent lentiviral shRNAs targeting RIPK1, FGFR4, SPHK1, AURKB and PIK3CD. mRNA expression was determined by qPCR 72 h after transduction. Cell viability was measured by CellTiter-Glo assay 5 day after transduction. Bars represent means ± s.d of at least three independent experiments. (*) indicates statistical significance compared with control cells transduced with a non-targeting shRNA (NS) ($P < 0.01$, Student's *t*-test).

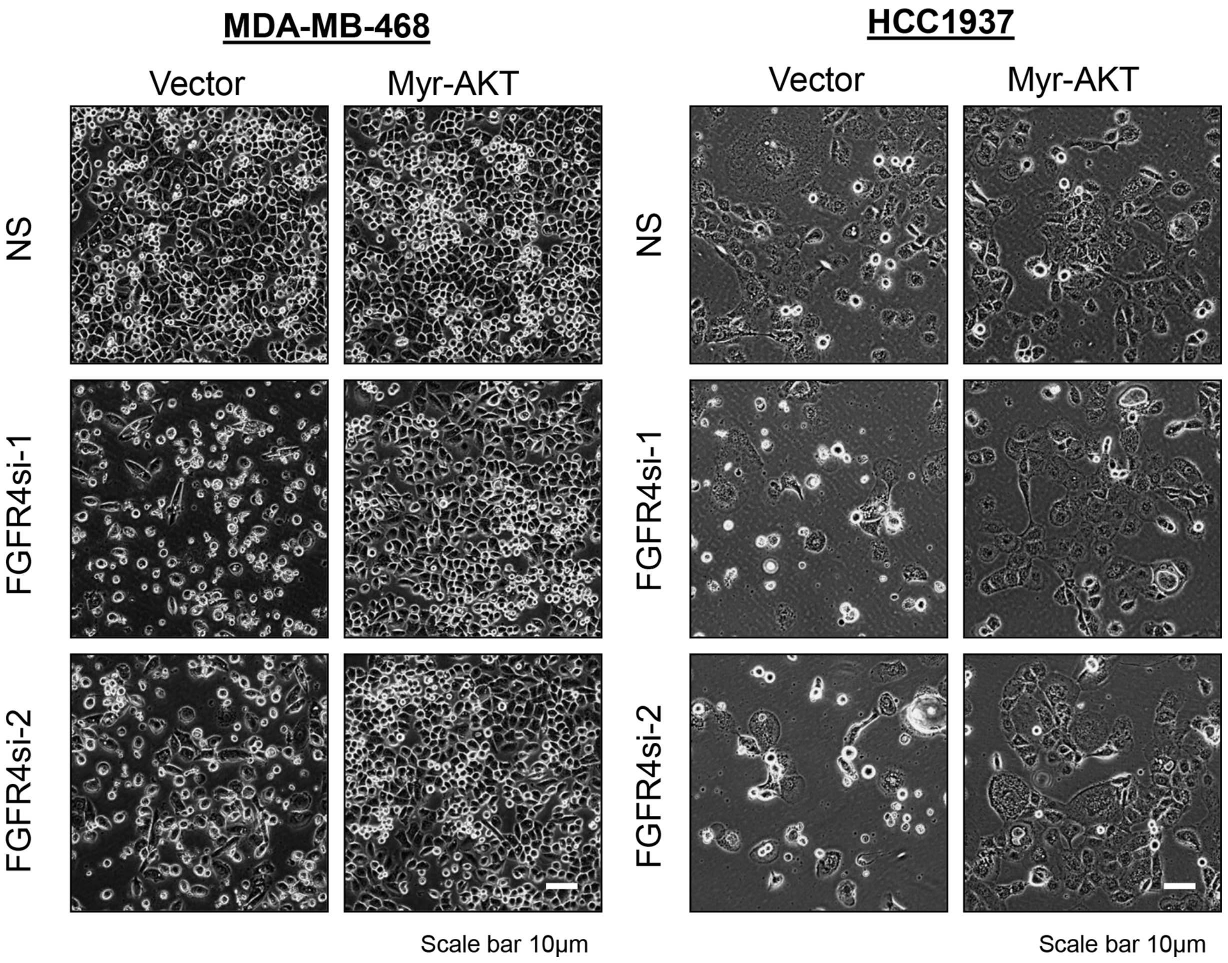

Supplementary Figure S2: Ectopic expression of myristoylated AKT rescues cell death induced by FGFR4 depletion. Cells were treated as in Figure 4. Cell morphology was recorded at 100X magnification 72 h post-transfection.

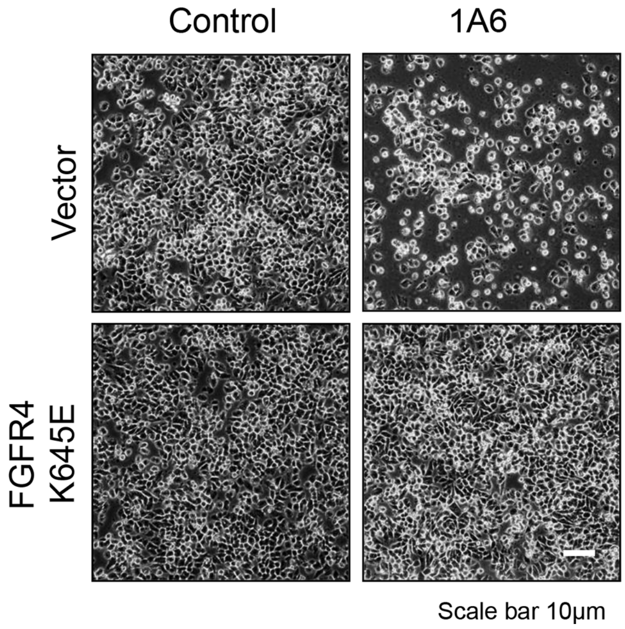

Supplementary Figure S3: The apoptotic effect of 1A6 is dependent on inhibition of FGFR4/FGF19 signaling. Cells were transfected with vector or constitutively active FGFR4 K645E mutant for 24 h followed by treatment with 10 μg/mL of 1A6 for 72 h. Morphological changes were recorded at 100X magnification.

Supplementary Table S1: FGFR4 expression is not associated with Gly388 SNP in human breast cell lines

Cell lines	mRNA[1]	Protein[2]	FGFR4 allele[3]
MDA-MB-468	25.05 ± 3.19	Positive	Gly/Gly
MDA-MB-231	12.55 ± 1.03	Negative	Gly/Arg
HCC1937	70.87 ± 5.17	Positive	Gly/Gly
SKBR3	268.03 ± 19.55	Positive	Arg/Arg
T47D	2378.54 ± 580.25	Negative	Gly/Arg
MCF-7	143.38 ± 6.54	Positive	Gly/Arg
MCF-10A	1.00 ± 0.15	Negative	Gly/Gly
HMEC	0.03 ± 0.01	Negative	n.d.

[1]FGFR4 mRNA levels was determined using qPCR and normalized to GAPDH.
[2]FGFR4 protein expression as detected by immunoblotting.
[3]FGFR4 alleles were determined using cDNA Sanger sequencing. n.d., not determined. Amplification of cDNA fragment was performed using Expand High Fidelity PCR System (Roche Diagnostic, IN, USA), according to manufacturer′s protocol. PCR products were analyzed on 2% agarose gel prior to Sanger sequencing. The forward and reverse primers used in cDNA amplification and DNA sequencing are 5′-GTGCTGCCAGAGGAGGAC-3′ and 5′-GACTCCAGGGAGAACTGTCG-3′ respectively.

Supplementary Table S2: Primer sequences for qPCR

Gene	Forward primer	Reverse primer
FGFR4	5′-ACCCCCAGCAAGCACCCTACTGG-3′	5′-GCTGCCCACAGCGTTCTCTACCA-3′
FGF19	5′-TGCACTACGGCTGGGGCGA-3′	5′-CATGGGCAGCATGGGCAGGA-3′
AURKB	5′-GGGCGGCCGGGAGAGTAGCA-3′	5′-AGTGCCGCGTTAAGATGTCGGGT-3′
RIPK1	5′-AGCTCGGCCGACATTTCCTGGCAT-3′	5′-TGGGCTGCTGTTTCGTCTGCCTGT-3′
SPHK1	5′-TGCACGAGGTGGTGAACGGGC-3′	5′-AGCCCCAGGCCAGGCTGAGC-3′
PIK3CD	5′-AGCTGCGCCGGGACGATAAGGA-3′	5′-TTGCTGCTCCGCTGTCTGGTTG-3′
GAPDH	5′-GTCTCCTCTGACTTCAACAGCG-3′	5′-ACCACCCTGTTGCTGTAGCCAA-3′

Supplementary Table S3: shRNAs target sequences

shRNAs	Target Sequence
FGFR4si-1	5′-GCCGACACAAGAACATCATCA-3′
FGFR4si-2	5′-ATCTACCTCTCGACCCACTAT-3′
RIPK1si-1	5′-CAGGCCAATTCCAAGTCATAT-3′
RIPK1si-2	5′-AGGTCATGTTCTTTCAGCTTA-3′
SPHK1si-1	5′-GCAGCTTCCTTGAACCATTAT-3′
SPHK1si-2	5′-GCAGGCATATGGAGTATGAAT-3′
AURKBsi-1	5′-CCTGCGTCTCTACAACTATTT-3′
AURKBsi-2	5′-GAAGAGCTGCACATTTGACGA-3′
PIK3CDsi-1	5′-GACCCAGAAGTGAACGACTTT-3′
PIK3CDsi-2	5′-GCACAGCGACAACATCATGAT-3′
